# De novo variants in *H3-3A* and *H3-3B* are associated with neurodevelopmental delay, dysmorphic features, and structural brain abnormalities

**DOI:** 10.1038/s41525-021-00268-8

**Published:** 2021-12-07

**Authors:** Volkan Okur, Zefu Chen, Liesbeth Vossaert, Sandra Peacock, Jill Rosenfeld, Lina Zhao, Haowei Du, Emily Calamaro, Amanda Gerard, Sen Zhao, Jill Kelsay, Ashley Lahr, Chloe Mighton, Hillary M. Porter, Amy Siemon, Josh Silver, Shayna Svihovec, Chin-To Fong, Christina L. Grant, Jordan Lerner-Ellis, Kandamurugu Manickam, Suneeta Madan-Khetarpal, Shawn E. McCandless, Chantal F. Morel, G. Bradley Schaefer, Elizabeth M. Berry-Kravis, Ryan Gates, Natalia Gomez-Ospina, Guixing Qiu, Terry Jianguo Zhang, Zhihong Wu, Linyan Meng, Pengfei Liu, Daryl A. Scott, James R. Lupski, Christine M. Eng, Nan Wu, Bo Yuan

**Affiliations:** 1 Department of Molecular and Human Genetics, Baylor College of Medicine, Houston, TX 77030 USA; 2 Baylor Genetics Laboratories, Houston, TX 77021 USA; 3 Department of Orthopedic Surgery, Beijing Key Laboratory for Genetic Research of Skeletal Deformity, Key Laboratory of Big Data for Spinal Deformities, State Key Laboratory of Complex Severe and Rare Diseases, Peking Union Medical College Hospital, Peking Union Medical College and Chinese Academy of Medical Sciences, 100730 Beijing, China; 4 Graduate School of Peking Union Medical College, 100005 Beijing, China; 5 Medical Research Center, State Key Laboratory of Complex Severe and Rare Diseases, Peking Union Medical College Hospital, Peking Union Medical College and Chinese Academy of Medical Sciences, 100730 Beijing, China; 6 Department of Pediatrics, University of Rochester School of Medicine and Dentistry, Rochester, NY 14642 USA; 7 Texas Children’s Hospital, Houston, TX 77030 USA; 8 Section of Genetics and Metabolism, University of Arkansas for Medical Sciences, Little Rock, AR 72701 USA; 9 Department of Medical Genetics, Children’s Hospital of Pittsburgh of UPMC, Pittsburgh, PA 15224 USA; 10 Institute of Health Policy, Management and Evaluation, University of Toronto, Toronto, ON M5T 3M6 Canada; 11 Li Ka Shing Knowledge Institute, St. Michael’s Hospital, Unity Health Toronto, Toronto, ON M5B 1A6 Canada; 12 Department of Pathology and Laboratory Medicine, Mount Sinai Hospital, Sinai Health, Toronto, ON M5G 1X5 Canada; 13 Lunenfeld-Tanenbaum Research Institute, Sinai Health, Toronto, ON M5G 1X5 Canada; 14 Rare Disease Institute, Children’s National Hospital, Washington, DC 20010 USA; 15 Nationwide Children’s Hospital (NCH) and The Ohio State University College of Medicine Section of Genetic and Genomic Medicine, Columbus, OH 43205 USA; 16 The Fred A. Litwin Family Centre in Genetic Medicine, University Health Network and Mount Sinai Hospital, Toronto, ON M5T 3L9 Canada; 17 Department of Molecular Genetics, University of Toronto, Toronto, ON M5S 1A8 Canada; 18 Department of Pediatrics, University of Colorado Anschutz Medical Campus, and Children’s Hospital Colorado, Aurora, CO 80045 USA; 19 Department of Laboratory Medicine and Pathobiology, University of Toronto, Toronto, ON M5S 1A8 Canada; 20 Department of Medicine, University of Toronto, Toronto, ON M5S 1A8 Canada; 21 Departments of Pediatrics, Neurological Sciences, and Biochemistry, Rush University Medical Center, Chicago, IL 60612 USA; 22 Department of Pediatrics, Stanford University School of Medicine, Stanford, CA 94305 USA; 23 Human Genome Sequencing Center, Baylor College of Medicine, Houston, TX 77030 USA; 24 Department of Pediatrics, Baylor College of Medicine, Houston, TX 77030 USA; 25 Present Address: Seattle Children’s Hospital, Seattle, WA 98105 USA; 26 Present Address: Department of Laboratory Medicine and Pathology, University of Washington, Seattle, UW 98105 USA

**Keywords:** Genetics research, Neurodevelopmental disorders, Molecular medicine, Neurodevelopmental disorders, Genetic testing

## Abstract

The histone H3 variant H3.3, encoded by two genes *H3-3A* and *H3-3B*, can replace canonical isoforms H3.1 and H3.2. H3.3 is important in chromatin compaction, early embryonic development, and lineage commitment. The role of H3.3 in somatic cancers has been studied extensively, but its association with a congenital disorder has emerged just recently. Here we report eleven de novo missense variants and one de novo stop-loss variant in *H3-3A* (*n* = 6) and *H3-3B* (*n* = 6) from Baylor Genetics exome cohort (*n* = 11) and Matchmaker Exchange (*n* = 1), of which detailed phenotyping was conducted for 10 individuals (*H3-3A* = 4 and *H3-3B* = 6) that showed major phenotypes including global developmental delay, short stature, failure to thrive, dysmorphic facial features, structural brain abnormalities, hypotonia, and visual impairment. Three variant constructs (p.R129H, p.M121I, and p.I52N) showed significant decrease in protein expression, while one variant (p.R41C) accumulated at greater levels than wild-type control. One H3.3 variant construct (p.R129H) was found to have stronger interaction with the chaperone death domain-associated protein 6.

## Introduction

Histones are DNA-binding proteins constituting the building blocks of chromatin, i.e. the nucleosomes, and play an important role in the epigenetic regulation of chromatin compaction, gene transcription and other processes such as DNA damage repair. Each nucleosome is constructed from four core histones; H2A, H2B, H3, and H4, and a linker histone H1. Histone H3 consists of canonical isoforms H3.1 and H3.2, which can be replaced by multiple H3 variants such as H3.3 encoded by two genes *H3-3A* (also known as *H3F3A*) and *H3-3B* (also known as *H3F3B*) that are expressed in both embryonic and differentiated cells^1–3^.

The role of chromatin dysregulation in neurodevelopmental multisystemic disorders have been described by several studies^4–6^. Mutations in H3.3 genes have been implicated in human malignancies via promoting tumorigenesis by perturbing H3.3 chromatin-related epigenetic functions^7^. A female individual with global developmental delay, short stature, acquired microcephaly, hypotonia, hypoplasia of the corpus callosum and cerebellum, cortical visual impairment, atrial septal defect, and dysmorphic facial features was reported to have a de novo missense variant in *H3-3A*^8^. Recently, de novo missense variants in *H3-3A* and *H3-3B* have also been reported to be associated with neurodevelopmental delay and neurologic abnormalities in a cohort of 46 individuals^8,9^. Here, we report de novo missense and stop-loss variants in *H3-3A* (*n* = 6) and *H3-3B* (*n* = 6) in 12 individuals with neurodevelopmental disorders and multiple system abnormalities and a potentially perturbed interaction between one H3.3 mutant and the chaperone death domain-associated protein 6 (DAXX) using transiently transfected cell models.

## Results

### Molecular findings

**Fig. 1:**
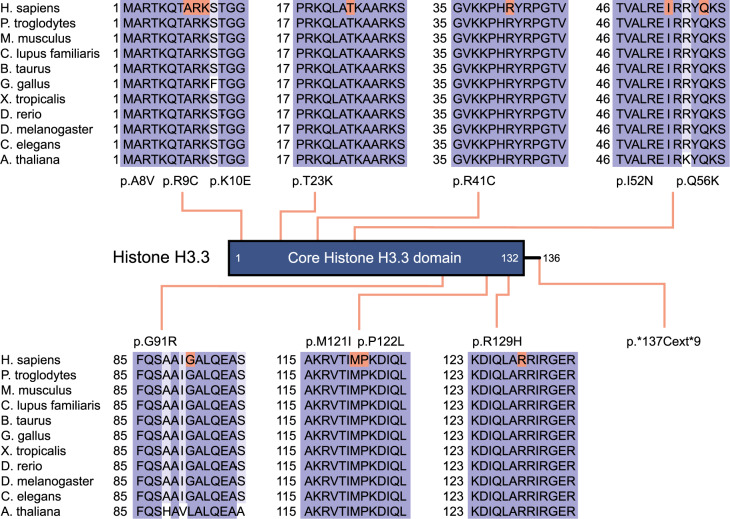
A two-dimensional schematic representation of histone H3.3. The main functional domain is shown with lines indicating the positions of variants. Protein sequence alignments of histone H3.3 with its orthologs are displayed.

All but one individuals were found to have de novo missense variants in evolutionarily highly conserved amino acids encoded by either *H3-3A* or *H3-3B* (Fig. 1), while the remaining individual (Individual 10) was found to have a de novo stop loss variant in *H3-3B*, which is predicted to result in incorporation of additional nine amino acids to the wild type protein (Table 1). All but one variants have not been observed in gnomAD (v2.1 and v3.0)^10^ and TOPMed (Freeze 8) databases^11^, while the c.386G>A (p.(R129H)) variant in *H3-3A* of Individual 4 was observed once in TOPMed Freeze 8. In silico prediction scores of the variants are in favor of pathogenicity (Table 1)^12–15^. Both genes encode a protein with identical polypeptide sequences forming five helices and eight beta strands that are involved in protein–protein interactions, and eight of the eleven missense variants are located in helices.

**Table 1:** Molecular characteristics of de novo *H3-3A* and *H3-3B* variants identified in individuals with neurodevelopmental disorders.

Gene	RefSeq ID	Genomic change (hg19)	Nucleotide change	Amino acid change	CADD	MPC	M-CAP	GERP_RS
*H3-3A*	NM_002107.7	NC_000001.10:g.226252173C>T	c.121C>T	p.R41C	26.2	2.37	0.045	4.32
		NC_000001.10:g.226253394C>A	c.166C>A	p.Q56K	25.7	2.36	0.135	4.97
		NC_000001.10:g.226253499G>C	c.271G>C	p.G91R	28.4	2.3	0.06	5.08
		NC_000001.10:g.226259132G>A	c.363G>A	p.M121I	24.7	2.06	0.127	5.83
		NC_000001.10:g.226259134C>T	c.365C>T	p.P122L	24.2	2.34	0.24	5.83
		NC_000001.10:g.226259155G>A	c.386G>A	p.R129H	24.5	2.24	0.058	5.83
*H3-3B*	NM_005324.5	NC_000017.10:g.73775233G>A	c.23C>T	p.A8V	26.6	1.78	0.036	5.08
		NC_000017.10:g.73775231G>A	c.25C>T	p.R9C	25.1	2.23	0.042	4.09
		NC_000017.10:g.73775228T>C	c.28A>G	p.K10E	27	2.3	0.056	5.08
		NC_000017.10:g.73775188G>T	c.68C>A	p.T23K	34	3.56	0.023	5.08
		NC_000017.10:g.73775018A>T	c.155T>A	p.I52N	33	4.92	0.071	5.28
		NC_000017.10:g.73774676_73774677del	c.410_411del	p.*137Cext*9	NA	NA	NA	NA

*CADD* combined annotation-dependent depletion, *MPC* missense badness, PolyPhen-2, and Constraint (Regional missense constraint), *M-CAP* Mendelian clinically applicable pathogenicity score, *GERP_RS* genomic evolutionary rate Profiling_Reduction score.

### Clinical findings

**Fig. 2:**
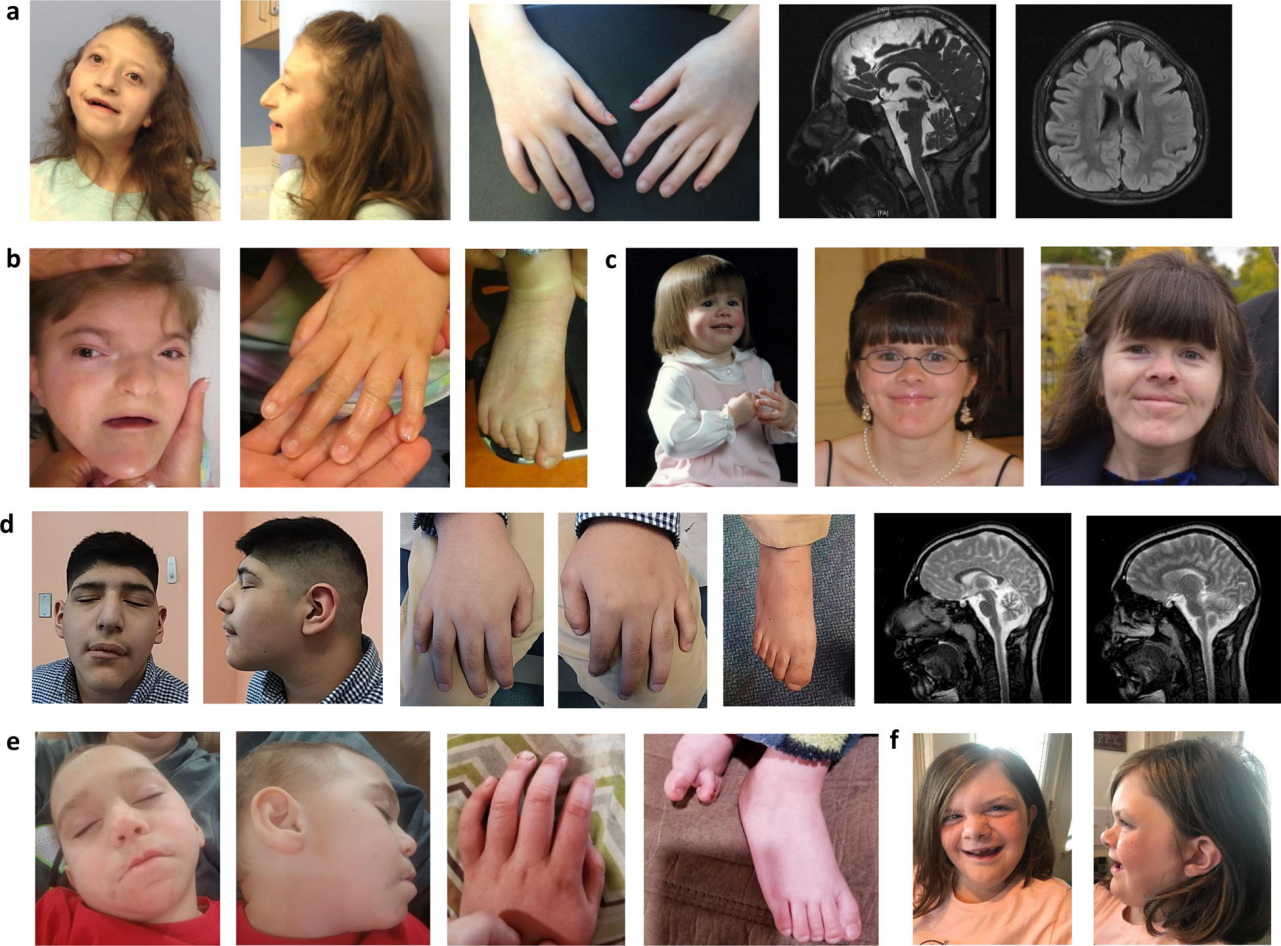
Images showing dysmorphic profiles involving facial features, hands and/or feet, and brain MRI images for available individuals. **a** Individual 1, **b** Individual 5, **c** Individual 6 (left to right: 20 months, 21 years, 33 years), **d** Individual 8, **e** Individual 9, and **f** Individual 10. Written informed consent form has been obtained from families for the publication of facial photos, and brain MRI images when applicable.

Clinical findings of 10 individuals with de novo *H3-3A* or *H3-3B* variants are summarized in Table 2 and Supplementary Table 1. All individuals had global developmental delays with gross motor, fine motor, and speech delays and intellectual disability. Short stature with or without failure to thrive were reported in eight (80%) individuals. Hypotonia (*n* = 8, 80%), gait difficulties (*n* = 7, 70%), and microcephaly (*n* = 6, 60%) were the most commonly reported neurological abnormalities, and seizures were reported in four (40%) individuals. Neurobehavioral abnormalities such as happy demeanor, autism, and stereotypic movements were also reported in five (50%) individuals. Although there is no emerging facial gestalt yet, dysmorphic facial features such as facial asymmetry, midface hypoplasia, thin upper lips, open mouth appearance, prognathia or pointed chin, and minor extremity abnormalities such as small hands and feet with fingertip pads were reported in eight (80%) individuals (Fig. 2). Four of seven (57%) individuals with brain MRI performed were reported to have structural brain abnormalities ranging from diminished white matter and hypomyelination to cortical dysplasia and leukoencephalopathy. Ophthalmologic, musculoskeletal, and gastrointestinal system problems were also reported in all individuals, similar to generally reported in other neurodevelopmental disorders. Two individuals also had congenital hypothyroidism.

**Table 2:** Clinical findings of individuals with de novo *H3-3A* and *H3-3B* variants.

Age and sex	Individual 1	Individual 2	Individual 3	Individual 4	Individual 5	Individual 6	Individual 7	Individual 8	Individual 9	Individual 10
	10 yo & Female	28 yo & Female	13 yo & Male	4.5 yo & Male	14 yo & Female	33 yo & Female	4yo & Female	12 yo & Male	5 yo & Male	8 yo & Female
Gene (Transcript)	*H3-3A* (NM_002107.6)				*H3-3B* (NM_005324.5)					
Variant	c.166C>A (p.Q56K)	c.271G>C (p.G91R)	c.365C>T (p.P122L)	c.386G>A (p.R129H)	c.23C>T (p.A8V)	c.25C>T (p.R9C)	c.28A>G (p.K10E)	c.68C>A (p.T23K)	c.155T>A (p.I52N)	c.410_411del (p.*137Cysext*9)
Prenatal	Unremarkable, uneventful	IUGR	Unremarkable, uneventful	Unremarkable, uneventful	NR	Induced for postterm pregnancy	Decreased fetal movements	SGA	NR	IUGR
Birth	Respiratory distress and difficulty feeding	NR	Unremarkable	Prematurity	SGA	Hypotonia Apneic episodes Difficulty feeding	Hypotonia, axial Weak cry Difficulty feeding	SGA	SGA	Possible seizure activity Immature thermoregulation
Growth/Endocrine	Short stature FTT	Short stature	Short stature FTT	Short stature with GH deficiency (delayed bone age [−3 to −4 SD]) FTT	Short stature FTT	Short stature Hypothyroidism Type 1 DM	None	Advanced bone age Precocious puberty Growth plateau at 11 years old	NR	Short stature Advanced bone age Congenital hypothyroidism
Neurodevelopmental	GDD (gross & fine motor and speech) Happy demeanor Water affinity	GDD (gross motor and speech) ID Happy demeanor	GDD (gross motor and speech)	GDD (gross & fine motor and speech) Happy demeanor	GDD/ID, non-verbal	GDD/ID (motor and speech delays)	GDD	GDD/ID (Motor and speech delays)	GDD Speech delay Developmental regression Autism	GDD Motor delay Speech delay Happy demeanor Stereotypic flying of arms
Neurologic	Microcephaly Hypotonia Wide-based gait Mild spasticity in the lower extremities	Microcephaly Hypotonia Hypertonia of the lower extremities Seizures Drooling Wide-based gait	Microcephaly Hypotonia Seizures Drooling Ataxia	Relative macrocephaly Hypotonia (R>L) Gait abnormality	Microcephaly Hypotonia Craniosynostosis?	Seizures, generalized	Hypotonia	Gait abnormality	Microcephaly Hypotonia Ataxia Seizures	Microcephaly Hypotonia Gait abnormality
Brain MRI	Moderately diminished white matter in the anterior halves of both cerebral hemispheres along with hypomyelination accompanied by ventriculomegaly	NR	NR	Borderline Macrocephaly	Leukoencephalopathy	Normal	Normal	Cortical dysplasia Cerebellar hypoplasia Hypoplasia of the corpus callosum	Normal	NR
Dysmorphic features	Facial asymmetry Hypoplastic helix Long and upslanting palpebral fissures Prognathia Long, thin fingers with camptodactyly Fingertip pads	Brachycephaly Synophrys Hypotelorism, mild Midface hypoplasia Open mouth appearance with full lips and protruding lower teeth Small hands & feet	None	Facial asymmetry (R>L) Slightly low-set ears Long eyelashes Open mouth appearance Pointed chin Bilateral branchial remnant (s/p) (more prominent on the right) Fingertip pads Small hands & feet	Plagiocephaly Heavy eyebrows Deep set eyes Long eyelashes Down-slanting palpebral fissures Thin upper lips Hypodontia Hypertrichosis in the trunk Sparse hair on the scalp Camptodactyly of 4^th^ and 5^th^ fingers	Flat facial profile Triangular shaped face Depressed nasal bridge Thin upper lip Relative mandibular prognathism Mild clubbing in fingers	Dolichocephaly Bitemporal narrowing Sparse hair on bitemporal areas Smooth philtrum, mild Incomplete single palmar crease Tapered fingers	Low anterior hairline Narrow forehead Large ears Down-slanting palpebral fissures Bulbous tubular nose Smooth philtrum Persistence of primary teeth Widely spaced teeth Pointed chin Bilateral 5^th^ finger clinodactyly and brachydactyly Bilaterally digitalized thumbs Short middle phalanges on his fingers Broad big toes Pes planus	NR	Prominent forehead Posteriorly rotated ears Prominent eyebrows Hemangioma over glabella Blue sclerae Down-slanting palpebral fissures Upturned nose Midface hypoplasia Thin upper lip Small mouth Micrognathia High arched palate
Visual	Esotropia	None	Strabismus	None	Retinal degeneration Dysplastic optic nerve	Strabismus	Nystagmus	Severe myopia (−18 to −20 diopter)	Astigmatism Blindness in the left eye	Esotropia
Musculoskeletal	Joint hypermobility Pes planus	Scoliosis	Joint hypermobility Pes planus	Mild asymmetry of the lower extremities (R>L) Pes planus, mild	Scoliosis	Scoliosis, thoracolumbar	NR	Joint contractures	Hypermobility	Scoliosis Hypermobility
Gastrointestinal	NR	Constipation	Constipation GERD	Unremarkable	NR	NR	Constipation	Constipation	Dysphagia Vomiting Congenial malformation of small bowel	NR
Cardiologic	None	None	None	Resolved pulmonary artery branch stenosis Resolved PACs	NR	NR	None	None	NR	Bicuspid aortic valve and partial fusion of aortic leaflets
Other clinical findings	NR	Teeth issues	Sleep dysregulation Reduced sweating Erythema multiforme	Delayed teeth eruption Chronic dysfunction of Eustachian tubes and adenoid hypertrophy	NR	NR	Sensorineural hearing loss, bilateral Laryngomalacia	Normal hearing	Recurrent otitis media Velvety and hyperextensible skin, patchy and soft hair	Shallow dental roots

*yo* years old, *IUGR* intrauterine growth retardation, *NR* not reported, *SGA* small for gestational age, *FTT* failure to thrive, *SD* standard deviation, *DM* diabetes mellitus, *GDD* global developmental delay, *ID* intellectual disability, *R* Right, L left, GERD gastroesophageal reflux disease, *PAC* premature atrial contractions.

### Functional characterization

**Fig. 3:**
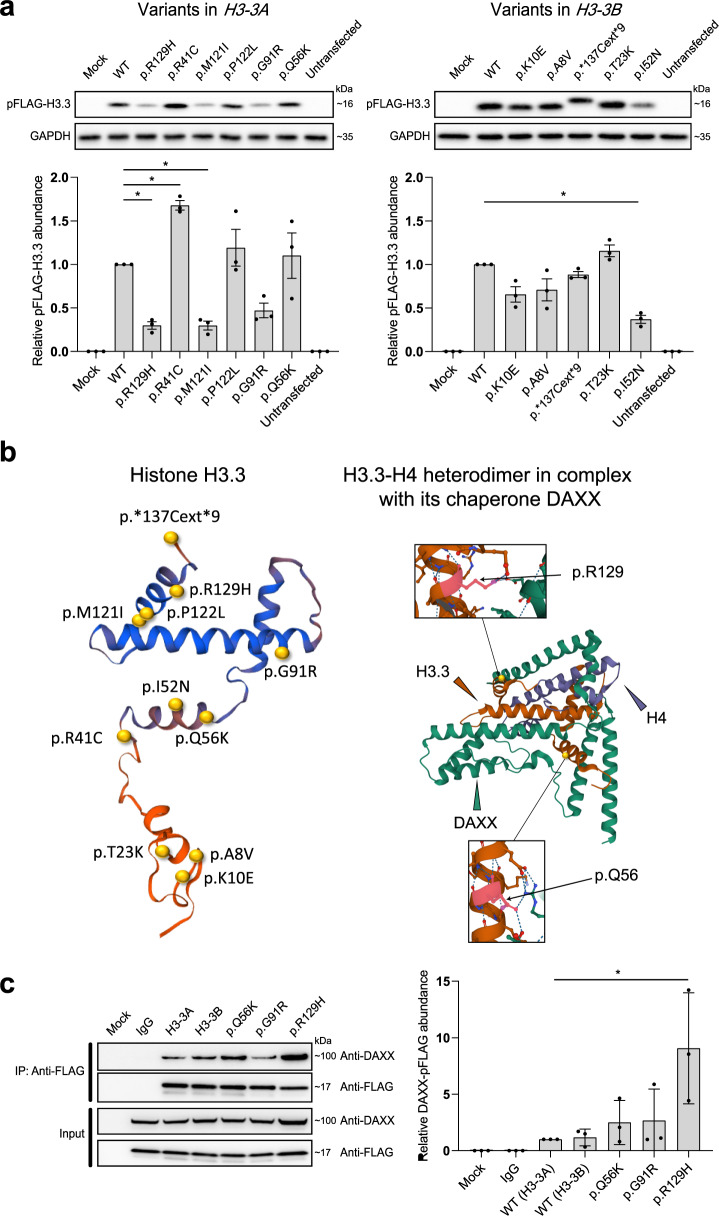
Functional consequences of the *H3-3A*/*H3-3B* variants. **a** Levels of mutant histone H3.3s in HEK293T cells. FLAG-tagged WT and mutant histone H3.3s were transfected into HEK293T cells. Protein levels in whole-cell lysates were detected by immunoblotting with anti-FLAG antibodies. GAPDH was used as a loading control. The abundance of FLAG relative to GAPDH was estimated by densitometry with the ImageJ Software 1.52 v. Plot presents mean ± SD of immunoblotting analysis from three independent HEK293T cell transfections. Statistical analysis was performed by unpaired Student’s *t* test, with *p < 0.001. **b** Visualization of the crystal structural of H3.3-H4 heterodimer in complex with its chaperone DAXX. Shown on the left is a crystal structure of histone H3.3 based on template structure SWISS-Model P84243, with the position of the identified variants presented in this study. The variants are indicated by yellow spheres. Shown on the right is an overview structure of the DAXX–H3–H4 complex based on template structure PDB:4HGA, with variants found in affected individuals (black arrows). The H3.3 monomer, H4 monomer, and DAXX are colored in orange, purple, and green, respectively. Histone H3.3 variants (p.Q56, p.R129) directly interact with DAXX, likely affecting their interaction. **c** Co-immunoprecipitation of FLAG-tagged H3.3s with DAXX in HEK293T cells. A FLAG-tagged mutant H3.3 and a DAXX were co-transfected into HEK293T cells. Cells expressing FLAG-tagged WT H3.3 and DAXX were used as a control. Co-immunoprecipitation was performed with nuclear proteins. The samples were derived from the same experiment or parallel experiments and the gels/blots were processed in parallel. The abundance of DAXX relative to FLAG was estimated by densitometry with the ImageJ Software 1.52v. Plot presents mean ± SD of immunoblotting analysis from three independent HEK293T cell transfections. Statistical analysis was performed by unpaired Student’s *t* test, with *p < 0.05. WT wild type, GAPDH glyceraldehyde 3-phosphate dehydrogenase, SD standard deviation, DAXX death domain-associated protein 6.

To determine the functional consequence of the *H3-3A*/*H3-3B* variants, we first attempted to determine protein levels of the 11 different mutants (one stop loss, ten missense) ascertained from the BG cohort in HEK293T cells. Mutagenesis was performed on cDNA clones in a pFLAG‐CMV‐4 expression vector using inverse PCR-based site-directed mutagenesis (see the “Methods” section). Each variant was incorporated into the corresponding gene (*H3-3A* or *H3-3B*) according to the variant–gene relationship in Table 1. Plasmids encoding FLAG-tagged wild-type (WT) and mutant H3.3s were transiently transfected into HEK293T cells. Anti-FLAG immunoblotting using whole-cell lysates of the transfected cells revealed a persistent lower protein level of three variants (p.R129H, p.M121I, and p.I52N). On the contrary, one variant (p.R41C) accumulated at greater levels than WT (Fig. 3a). Other variants did not show significantly altered protein levels. Notably, the c. 410_411del (p.*137Cext*9) variant in *H3-3B* resulted in an elongated protein with a similar protein level compared to WT (Fig. 3a). These results indicated differential impact of these variants on H3.3 protein abundance and stabilization in whole-cell lysates, while the exact mechanism for altered protein level is unclear.

Histone H3.3 interacts with histone H4 to form the H3.3–H4 heterodimer and cooperates with death domain-associated protein 6 (DAXX) to direct the deposition of H3.3 onto pericentric and telomeric heterochromatin^16–18^. Crystal structures of the DAXX–H3.3–H4 complexes (PDB:4HGA/4H9N/4H9O/4H9P/4H9Q) suggest that two residues (Q56 and R129) of H3.3 directly interact with DAXX by forming a hydrogen bond^16,19^. In addition, a previous study showed that the G91 in the ‘AAIG’ motif (A88, A89, I90 and G91) is a dominant contributor to chaperone specificity^19^. Therefore, the substitution of these residues with a different amino acid (in our cases p.Q56K, p.G91R and p.R129H) might affect protein–protein interaction with chaperones such as DAXX (Fig. 3b). To investigate this hypothesis, we co-transfected HEK293T cells with a FLAG-tagged mutant histone H3.3 (p.Q56K, p.G91R, and p.R129H, respectively) and a WT DAXX, and their association was analyzed by co-immunoprecipitation (Co-IP) of nuclear proteins. The Co-IP assays showed that p.R129H had a significantly stronger interaction with DAXX, suggesting that this variant might affect the deposition of histone H3.3 (Fig. 3c). To profile the intracellular distribution of histone H3.3 mutants, we next transfected FLAG-tagged mutants into HEK293T cells, and immunofluorescence was subsequently performed. The results revealed that both WT and mutants were found to be unaffected and localized inside the nucleus (Supplementary Figs. 1 and 2).

Since the N-terminal tail of the histone H3.3 is targeted for post-translational modifications (PTMs) to regulate protein activity, we further used various in silico prediction programs to explore the possible impact on PTMs imposed by mutations of H3.3 residues (Supplementary Methods). In silico predictions suggested that p.R9C, p.K10E, p.T23K, p.Q56K, and p.*137Cext*9 might alter the PTMs of histone H3.3, including phosphorylation, glycosylation, methylation, acetylation, and ubiquitination, while other mutants (p.G91R, p.M121I, p.P122L, p.R129H, p.A8V, and p.I52N) did not show apparent change on PTMs (Supplementary Table 2 and Supplementary Fig. 3). To facilitate understanding of various mechanisms of the reported variants, all effects of the variants from the experiments and in silico predictions are summarized in Supplementary Table 3.

## Discussion

Genes involved in chromatin regulation and histone remodeling have been implicated in many neurodevelopmental syndromes with multisystemic involvement^4–6^. Although the role of deleterious variants in the core histone proteins in human cancers have been studied extensively, particularly gliomas, chondrosarcomas, and giant cell tumors of the bone^20^, their effect on human development were not fully understood. Recently, individuals with neurodevelopmental problems carrying predicted pathogenic germline variants in *H3-3A* and *H3-3B* have been reported in the literature as well as deposited in ClinVar^8,21–24^.

The clinical findings of individuals carrying de novo *H3-3A* and *H3-3B* variants in this study overlap with those reported before and with chromatinopathies in general^25^. In a large series of 46 individuals, Bryant et al. reported global developmental delay in the majority of their individuals along with hypotonia (67%), seizures (50%), short stature (33%), microcephaly (26%), and brain anomalies on imaging (73%)^21^. A moderate-to-severe global developmental delay was also observed in all individuals in our study, which usually affects all three major domains of psychomotor development, along with hypotonia (80%), gait abnormalities (70%), microcephaly (60%), variable structural brain abnormalities (57%), and seizures (40%). While short stature is reported in only one third of individuals reported by Bryant et al., all but two individuals in our study were reported to have short stature with or without failure to thrive. Furthermore, four individuals in our study also had endocrine system abnormalities including hypothyroidism, type 1 diabetes mellitus, advanced bone age, and precocious puberty. Bryant et al. also reported hypothyroidism in two of their individuals^21^, which altogether may suggest the endocrine system as being a major affected system. Craniosynostosis/abnormal head shape was reported by Bryant et al. in 30% of their individuals, and four (40%) in our study were reported to have possible craniosynostosis, facial asymmetries, and brachycephaly. Additionally, as noted by Bryant et al. although the majority of the individuals had variable dysmorphic facial features, there is no emerging facial gestalt common to all affected individuals. In contrast, while progressive neurologic disease and atrial septal defect were reported by Bryant et al. in ~1/5 of their individuals, only two in our study were reported to have non-structural congenital heart disease, and none was reported to have progressive neurologic disease. These differences might be explained by the variability in the ages of the individuals and in the duration of follow-up time as well as by the severity of the cardiac defect that could be overlooked without performing echocardiogram in all individuals. Furthermore, other genomic and environmental factors can also contribute to this variation as individuals with the same variants in both studies manifest variable phenotypes. For example, while individuals with the same p.G91R variant in *H3-3A* in both studies (*n* = 2) had highly overlapping clinical findings, individuals with p.P122L variant in *H3-3A* (*n* = 2) and individuals with p.R9C variant in *H3-3B* (*n* = 2) differ between each other in terms of short versus tall stature, failure to thrive versus obesity, and presence of endocrine abnormalities. Seven of thirteen H3-3A variants reported by Kaplanis et al. were also reported by Bryant et al. and our study, however, the clinical findings of those individuals were not reported in the Kaplanis et al. study to allow clinical comparison. Future natural history and functional studies may both outline the full clinical spectrum in the affected individuals and allow genotype–phenotype correlations by also objectively evaluating the severity of neurodevelopmental delays in each individual.

Fifty of fifty-two (96%) variants reported so far are missense variants, consistent with the low missense variation seen in the individuals cataloged in gnomAD v2.1 (missense *Z*-scores = 3.16 and 2.88 for *H3-3A* and *H3-3B*, respectively). The remaining two stop loss variants reported in this paper and by Bryant et al. are not expected to result in nonsense-mediated decay (NMD). Consistent with the speculation of escaping NMD, western blot analysis of the variant in our study demonstrated a mutant protein with larger size and comparable abundance to the WT control. Three missense variants (p.G91R, p.M121I, and p.P122L) as well as allelic variants (p.R129C and p.T23I) affecting the same residues as in p.R129H and p.T23K of our study were also reported by Bryant et al., who showed the H3.3 proteins carrying those missense variants resulted in increased proportion of H3.3 in H3^21^. Furthermore, we performed a Co-IP assay and demonstrated that p.R129H variant significantly enhanced the binding of histone H3.3 to DAXX, which might disrupt heterochromatic modifications and lead to aberrant transcription^26^.

Alternatively, the whole histone protein can be swapped out for a histone variant, often highly similar in protein sequence but with a distinct functionality^27^. Although *H3-3A* and *H3-3B* contain introns as opposed to the canonical histone genes and are replication-independent for their expression, the encoded H3.3 protein sequence differs only by five amino acids from H3.1 and H3.2 in human cells. It was recently shown that there is an equilibrium between canonical (H3.1/H3.2) and non-canonical (H3.3) isoforms during embryonic development in mice via transcription-independent and replication-dependent manner^28^. Additionally, Strobino et al. reported that loss of histone H3.3 homolog HIS-72 in *C. elegans* results in DNA replication defects^29^. Thus, the disruption of the equilibrium in the ratio of subunit components of H3 in the developing embryo might result in differential epigenetic modifications and replication defects that might have adverse cellular and developmental outcomes.

Histone proteins are subject to numerous posttranslational modifications, which among other functions serve as binding locations for chaperone proteins and transcription factors promoting or repressing transcription depending on the type of modification and its exact location. H3.3 has been shown to have binary interactions with at least 10 proteins including DAXX, DNMT3A, ZMYND11, and SETD2. These interactions take place at different residues for each interaction, and different variants might disrupt interactions with certain proteins. However, the associated neurodevelopmental phenotype reported to-date is highly consistent among all variant carriers with variable expression of the phenotype to some extent. This suggests a global unifying underlying mechanism for all disrupting variants that warrants further investigation. The unique protein–protein interaction disruptions might explain the variability in the extent of the phenotype.

Interestingly, none of the individuals reported so far has had any type of cancer, although the variants previously implicated in somatic cancer development were reported by Bryant et al., and we showed enhanced binding of H3.3 protein composed of mutated H3-3A isoform to DAXX which was also implicated in molecular mechanism of H3-3A-mediated cancer development^20^. Separate mechanisms might be speculated to underlie the tumorigenesis and disruption of normal embryonic development in humans. For example, variants affecting the lysine residues directly (such as K9M, K27M, and K36M) or indirectly (such as G34R) have been shown to perturb the methylation of corresponding lysine residues in cancers (see Review by Lowe et al. 2019)^20^. On the other hand, cancer development is also uncommon in individuals with neurodevelopmental disorders carrying pathogenic germline variants in the methyl-/demethyltransferases such as *EHMT1* (Kleefstra Syndrome 1, MIM:610253), *KMT2A* (Wiedemann–Steiner syndrome, MIM:605130), and *KDM4B*^30^ that regulate methylation of those lysine residues in H3 protein.

In summary, we report de novo missense and stop-loss variants in *H3-3A* and *H3-3B* in 12 unrelated individuals with overlapping neurodevelopmental phenotypes. In silico predictions suggest modified patterns of PTMs for the H3.3 proteins with mutations affecting the N-terminal tail. In vitro studies using transiently transfected cell line model suggest differentially altered protein level and/or stronger protein-protein interaction with chaperone DAXX resulted from the H3.3 variants reported in this study. Further studies are warranted to uncover the underlying molecular mechanism of H3.3 defects.

## Methods

### Ascertainment

Eleven individuals with de novo variants in *H3-3A* or *H3-3B* were identified by retrospective review of ~16,000 individuals referred for clinical exome sequencing (ES) at Baylor Genetics Laboratories (BG) between December 2011 and December 2019 with a range of indications including multiple congenital anomalies and neurodevelopmental disorders. All individuals involved in the study provided informed consents for clinical ES. Inclusion of anonymized individuals with minimum clinical information as part of an aggregated study based on retrospective review of data from BG clinical laboratories was approved by the Institutional Review Board (IRB) of Baylor College of Medicine (BCM, IRB# H-41191). The twelfth individual (Individual 6) was identified through Matchmaker Exchange, thus only clinical and molecular genetics data were presented in the study^31^. Among the twelve individuals, ten were subsequently recruited into a research study to provide case-level detailed clinical information. These individuals were enrolled by obtaining written informed consents approved by the IRB of BCM (IRB# H-22769). The authors affirm that human research participants provided informed consent for publication of images in Fig. 2.

### ES analysis

ES analysis was performed at BG as previously described^32^. Samples were also concurrently analyzed by SNP arrays (Illumina HumanExome-12 or CoreExome-24 array) for quality control of the ES data, as well as for detecting large copy-number variants (CNVs) and regions of absence of heterozygosity (AOH)^33,34^. Homozygous/hemizygous deletions were also analyzed using an in-house developed pipeline based on exome read-depth analysis as previously described^35^. The ES-targeted regions cover >23,000 genes for capture design (VCRome by NimbleGen^®^), including both coding and untranslated region exons. The mean coverage of target bases was >100×, and >95% of target bases were covered at >20×^32^. PCR amplification and Sanger sequencing was performed to verify all candidate variants in the probands according to standard procedures. Of note, reanalysis of ES data for individuals who had their first ES analysis prior to January 2020 was performed as described recently^36^ to evaluate for the presence of other potentially causative variants. No other potential molecular diagnoses contributed by other loci were identified by the reanalyses.

### Plasmid construction

A full-length cDNA clone of human *H3-3A* and *H3-3B* (GenBank: NM_002107.7 and NM_005324.5) was amplified using polymerase chain reaction (PCR). The PCR amplicons were cloned into the *NotI* and *BglII* sites of the pFLAG‐CMV‐4 expression vector (Sigma‐Aldrich). Inverse PCR‐based site‐directed mutagenesis was performed using KOD‐Plus‐Neo Kits (KOD-401, Toyobo) according to the manufacturer’s instructions. Mutated plasmid was used to transform *E. coli* clones and validated by Sanger sequencing. The primers used in mutagenesis are provided in Supplementary Table 4.

### Transient transfection of HEK293T cells and immunoblotting analysis

A HEK293T cell line was a gift from the Institute of Basic Medical Sciences, Chinese Academy of Medical Sciences. Cells were cultured in Dulbecco’s modified Eagle medium (C11995500BT, Thermo Fisher) containing 10% fetal bovine serum (10099141c, Thermo Fisher) and 1% penicillin–streptomycin (15140122, Thermo Fisher). Cells seeded at 1.5 × 10^6^ cells/well in six-well plates were transfected with plasmids (5.0 ug) encoding WT or mutant N-terminal FLAG-tagged H3.3s using Lipofectamine 3000 Transfection Reagent (L3000015, Thermo Fisher). The efficiency was examined by transfecting cells with eGFP‐pFLAG‐CMV-H3.3 plasmid. After a 24-h transfection, whole cell protein was extracted using RIPA Lysis and Extraction Buffer (89900, Thermo Fisher). SDS–PAGE and immunoblotting analysis was performed by standard methods. Anti‐DDDDK tag antibody (1:1000, ab1162, Abcam), binds to FLAG^®^ tag sequence, and anti-GAPDH monoclonal antibody (1:4500, K200057M, Solarbio) were used as the primary antibodies. Peroxidase AffiniPure goat anti-rabbit IgG (H + L) (1:20,000, 111-035-003, Jackson ImmunoResearch) and peroxidase AffiniPure goat anti-mouse IgG (H + L) (1:20000, 115-035-003, Jackson ImmunoResearch) were used as the secondary antibodies. Immunoblot images were analyzed using Image Lab Software 3.0 (BioRad). The abundance of FLAG relative to GAPDH was estimated by densitometry with the ImageJ Software 1.52v. All experiments were performed in triplicate.

### Co-transfection of DAXX with histone H3.3 mutants and co-IP

Plasmids encoding a WT or mutant N-terminal FLAG-tagged H3.3 (15.0 μg) and a WT DAXX (15.0 μg) were transiently co-transfected into HEK293T cells cultured in 10-cm dishes using Lipofectamine 3000 transfection reagent (L3000015, Thermo Fisher). After a 48-h transfection, nuclear proteins were extracted using NE-PER™ Nuclear and Cytoplasmic Extraction Reagents (78833, Thermo Fisher) with Halt™ Protease Inhibitor Cocktail (87786, Thermo Fisher). Dynabeads Protein G (40 μl, 1003D, Thermo Fisher) was incubated with anti-DDDDK antibody (6.0 μg, M185-3L, MBL International), binds to FLAG^®^ tag sequence, for 4 h at room temperature. The Dynabeads protein G–Ig complexes were then incubated in 0.2 M triethanolamine with 20 mM dimethylpyrimidine (DMP)–2HCl for 30 min at room temperature with end-over-end rotation. After the crosslinking, the beads were mixed with nuclear proteins and incubated overnight at 4 °C with end-over-end rotation, followed by three washes with lysis buffer. Bound target proteins were then eluted by heating the beads for 10 min at 70 °C in SDS loading buffer (NP0007, NP0009, Thermo Fisher) and subjected to Western blotting. Anti-DDDDK antibody (1:1000, 20543-1-AP, Proteintech), binds to FLAG^®^ tag sequence, and anti-DAXX antibody (1:5000, ab32140, Abcam) were used as the primary antibodies. Goat Anti-Rabbit IgG (H + L) (1:5000, ZB-2301, ZSGB-BIO) was used as the secondary antibody. The abundance of DAXX relative to FLAG was estimated by densitometry with the ImageJ Software 1.52v.

### Immunofluorescence

The HEK293T cells were implanted on glass slides in 24‐cell plates and transfected with plasmids encoding WT or mutant N-terminal FLAG-tagged H3.3s (0.5 μg) using Lipofectamine 3000 Transfection Reagent (L3000015, Thermo Fisher). After a 24-h transfection, the glass slides were rinsed with phosphate‐buffered saline (PBS) and fixed in 4% formaldehyde (P1110, SolarBio). The fixed cells were then rinsed with PBS four times and blocked with PBS containing 25% goat serum and 0.3% Triton™ X‐100 (Sigma‐Aldrich) for 1 h. Anti‐DDDDK tag antibody (P01L072; GENE-PROTEIN LINK), binds to FLAG^®^ tag sequence, was used as the primary antibody. Goat anti‐rabbit IgG (H + L) cross‐adsorbed secondary antibody and Cyanine 3 (A10520; Thermo Fisher) were used as the secondary antibodies. Dyed slides were mounted using VECTASHIELD mounting medium with DAPI (H-1200-10, Vector Laboratories). Fluorescence was visualized using a confocal microscopy (Nikon Eclipse Ti2).

### In silico prediction of PTMs of histone H3.3

PTM sites, including phosphorylation, glycosylation, methylation, acetylation, and ubiquitination, were predicted using KinasePhos 2.0^37^, GPP^38^, GPS-MSP^39^, PAIL^40^, and NR-2L^41^, respectively. The full-length amino acid sequences of WT or mutant H3.3s were inputted into the website servers to predict modified sites using default settings. The PTM sites provided by the computational approaches were then located on the amino acid sequences.

### Statistical analysis

The one-tailed unpaired Student’s *t* test was used for statistical analysis of immunoblotting results. Statistical analyses were performed with SPSS version 21.0 and GraphPad Prism version 8.0.2.

### Reporting summary

Further information on research design is available in the Nature Research Reporting Summary linked to this article.

## Supplementary information

Supplementary Information

Reporting Summary

## Data Availability

The main data supporting the findings of this study are available within the paper and its Supplementary information. Reasonable request of additional experimental results and/or materials is feasible and is conditioned to protect research participants’ privacy. Most materials and reagents used in this study are commercially available. Families enrolled in this study did not provide additional consent to share raw dataset for exome sequencing and SNP array in a public repository. The reported variants have been submitted to ClinVar under the accession numbers from SCV001759938 to SCV001759949.
