# Supplementary material for: De novo variants in H3-3A and H3-3B are associated with neurodevelopmental delay, dysmorphic features, and structural brain abnormalities
Source: NPJ Genom Med. 2021 Dec 7;6:104. doi: 10.1038/s41525-021-00268-8 (PMC8651650; doi:10.1038/s41525-021-00268-8)

Supplementary Data

De novo variants in *H3-3A* and *H3-3B* are associated with neurodevelopmental delay, dysmorphic features, and structural brain abnormalities

Supplementary Table 1. Detailed clinical findings of subjects with *de novo H3-3A* and *H3-3B* variants.

		Individual 1	Individual 2	Individual 3	Individual 4	Individual 5	Individual 6	Individual 7	Individual 8	Individual 9	Individual 10
**Age & Sex**		10 yo, Female	28 yo, Female	13 yo, Male	4.5 yo, Male	14 yo, Female	33 yo, Female	4 yo & Female	12 yo, Male	5 yo, Male	8 yo, Female
**Gene**		*H3-3A*				*H3-3B*					
**Transcript**		NM_002107.7				NM_005324.5					
**Variant (nucleotide)**		c.166C>A	c.271G>C	c.365C>T	c.386G>A	c.23C>T	c.25C>T	c.28A>G	c.68C>A	c.155T>A	c.410_411del
**Variant (protein)**		p.Q56K	p.G91R	p.P122L	p.R129H	p.A8V	p.R9C	p.K10E	p.T23K	p.I52N	p.*137Cysext*9
**Literature report**		NA	PMID:33268356	PMID:33268356	NA	ClinVar VCV000521247.1	PMID:33268356	NA	NA	NA	NA
**Prenatal**		Unremarkable, uneventful	IUGR	Unremarkable, uneventful	Unremarkable, uneventful	NR	Unremarkable, uneventful	Decreased fetal movement	SGA	NR	IUGR
**Birth**		Respiratory distress and difficulty feeding	NR	Uneventful	Prematurity	SGA	Induced for post-term<br>Hypotonia<br>Apneic episodes<br>Difficulty feeding	Hypotonia, axial<br>Weak cry<br>Difficulty feeding	SGA	SGA	Possible seizure activity<br>Immature thermoregulation
**Growth/En**	**Short stature**	+	+	+	+, GH deficiency	+	+	-	Growth plateau at 11 years old	NR	+
	**Bone age**	NR	NR	NR	delayed	NR	-	NR	advanced	NR	advanced
	**FTT**	+	-	+	+	+	-	-	-	NR	NR
**Neurodevelopmental**	**GDD**	+	+	+	+	+	+	+	+	+, regression	+
	**Motor delay**	gross & fine	gross	+	gross & fine	+	gross & fine	+	+	+	+
	**Speech delay**	+	+	+	+	+, non-verbal	+	+	+	+	+
	**ID**	NR	+	+	NR	+	+	+	+	NR	NR
	**Happy demeanor**	+	+	-	+	NR	-	-	NR	NR	+
	**Other behavior problems**	Water affinity	NA	-	NA	NA	-	NR	NA	Autism	Stereotypic arm flapping
	**Microcephaly**	+	+	-	-, Relative macrocephaly	+	-	-	-	+	+
	**Hypotonia**	+	+	+	+	+	+	+	NR	+	+
	**Gait abnormality**	+, wide-based	+, wide-based	+, ataxia	+	NR	-	-	+, impaired balance	+,ataxia	+
	**Seizures**	NR	+	+	-	NR	+	-	-	+	NR
	**Visual**	Esotropia	NR	Strabismus	NR	Retinal degeneration,	Strabismus	Nystagmus	Severe myopia (-18 to -20 diopter)	Astigmatism<br>Blindness in the left eye	Esotropia

						Dysplastic optic nerve					
	**Other neurological features**	Mild spasticity in the lower extremities	Hypertonia of the lower extremities, Drooling	Drooling	NA	NA	NA	NA	NA	NA	NA
**Dysmorphic features**	**Facial asymmetry**	+	-	-	+	-	-	-	-	NR	-
	**Ear**	Hypoplastic helix	-	-	Low-set	-	-	-	Large	Hyperextensible	Posteriorly rotated
	**Midface hypoplasia**	-	+	-	-	-	+	-	-	NR	+
	**Palpebral fissures**	Long and upslanting	-	-	-	Down-slanting	-	-	Down-slanting	NR	Down-slanting
	**Forehead**	-	-	-	-	-	-	Narrow	Narrow	NR	Prominent
	**Thick eyebrows**	-	-	-	-	+	-	-	-	NR	+
	**Eye**	-	Hypotelorism, mild	-	-	Deep-set eyes	-	-	-	NR	Blue sclerae
	**Mouth**	-	Open mouth appearance with full lips and protruding lower teeth	-	Open mouth appearance	Thin upper lips	Thin upper lip	Smooth philtrum	Smooth philtrum	NR	Small mouth, Thin upper lip, High arched palate
	**Nose**	-	-	-	-	NR	Depressed nasal bridge	-	Bulbous tubular nose	NR	Upturned nose
	**Hair**	NR	Synophrys	-	Long eyelashes	Long eyelashes, Hypertrichosis on the trunk, Sparse hair on the scalp	-	Sparse hair on bitemporal areas	Low anterior hairline	Patchy and soft	NR
	**Extremity**	Long, thin fingers with camptodactyly, Fingertip pads	Small hands & feet	-	Fingertip pads, Small hands & feet, Mild asymmetry of the lower extremities (R>L)	Camptodactyly of 4th and 5th fingers	Mild clubbing in fingers	Tapered fingers Incomplete single palmar creases	Bilateral 5th finger clinodactyly and brachydactyly, Bilaterally digitalized thumbs, Short middle phalanges on his fingers, Broad big toes	NR	NR
	**Teeth**	-	Teeth issues	-	Delayed teeth eruption	Hypodontia	-	-	Wide spaced, Persistence of primary teeth	NR	Shallow roots

	**Jaw**	Prognathism	-	-	Pointed chin	-	Relative mandibular prognathism	-	Pointed chin	NR	Micrognathia
**Musculoskelet**	**Scoliosis**	NR	+	-	NR	+	+	-	NR	NR	+
	**Pes planus**	+	NR	+	+	NR	-	-	+	NR	NR
	**Joint**	hypermobility	NR	Hypermobility	NR	Contractures	-	-	NR	Hypermobility	Hypermobility
**Gastrointestin**	**Constipation**	NR	+	+	NR	NR	-	+	+	NR	NR
	**Other Gastrointestinal features**	NA	NA	GERD	NA	NA	-	-	NA	Dysphagia, Vomiting, Congenial malformation of small bowel	NA
**Brain MRI**		Moderately diminished white matter in the anterior halves of both cerebral hemispheres along with hypomyelination accompanied by ventriculomegaly	NR	Normal	Borderline Macrocephaly	Leukoencephalopathy	Normal	Normal	Cortical dysplasia, Cerebellar hypoplasia Hypoplasia of the corpus callosum	Normal	NR
**Cardiac**		-	-	NA	Resolved pulmonary artery branch stenosis, Resolved PACs	NR	-	-	-	NR	Bicuspid aortic valve and partial fusion of aortic leaflets
**Other clinical findings**		NR	Brachycephaly	Sleep dysregulation, reduced sweating, Erythema multiforme	Bilateral branchial remnant (s/p) (more prominent on the right), Chronic dysfunction of Eustachian tubes and adenoid hypertrophy	Plagiocephaly	Hypothyroidism Type 1 DM	Bilateral sensorineural hearing loss Laryngomalacia	Precocious puberty	Recurrent otitis media	Hemangioma over glabella, Congenital hypothyroidism

Abbreviations: +, present; -, not present; yo, years old; NA, Not available; IUGR, Intrauterine growth retardation; NR, Not reported; SGA, Small for gestational age; GH, Growth hormone; FTT, Failure to thrive; GDD, Global developmental delay; ID, Intellectual disability; R, Right; L, Left; GERD, gastroesophageal reflux disease; PAC, Premature atrial contractions; s/p, status/post; DM, Diabetes mellitus.

Supplementary Table 2. *In silico* prediction of post translational modifications of H3.3 mutants.

Gene	Variant	Phosphorylation[1]	Glycosylation[2]	Methylation[3]	Acetylation[4]	Ubiquitination[5]
H3-3A	p.R41C	-	-	-	-	-
	p.Q56K	-	-	-	New acetylation site	-
	p.G91R	-	-	-	-	-
	p.M121I	-	-	-	-	-
	p.P122L	-	-	-	-	-
	p.R129H	-	-	-	-	-
H3-3B	p.A8V	-	-	-	-	-
	p.R9C	-	-	Loss of methylation	-	New ubiquitination site
	p.K10E	-	-	Loss of methylation	Loss of acetylation	Loss of ubiquitination and New ubiquitination site
	p.T23K	Loss of phosphorylation	Loss of glycosylation	-	New acetylation site	-
	p.I52N	-	-	-	-	-
	p.*137Cext*9	New phosphorylation site	-	-	-	-

Abbreviations: -, *in silico* predictions do not show change on PTMs.

Supplementary Table 3. Effects of the variants from the experiments and in silico predictions

**Gene**	**RefSeq ID**	**Nucleotide change**	**Amino acid change**	**Decreased protein level of whole-cell lysates**	**Enhanced interaction with DAXX**	**Predicted altered post-translational modifications**
H3-3A	NM_002107.7	c.121C>T	p.R41C	✓		
	NM_002107.7	c.166C>A	p.Q56K			✓
	NM_002107.7	c.271G>C	p.G91R			
	NM_002107.7	c.363G>A	p.M121I	✓		
	NM_002107.7	c.365C>T	p.P122L			
	NM_002107.7	c.386G>A	p.R129H	✓	✓	
H3-3B	NM_005324.5	c.23C>T	p.A8V			
	NM_005324.5	c.25C>T	p.R9C			✓
	NM_005324.5	c.28A>G	p.K10E			✓
	NM_005324.5	c.68C>A	p.T23K			✓
	NM_005324.5	c.155T>A	p.I52N	✓		
	NM_005324.5	c.410_411del	p.*137Cext*9			✓

Abbreviations: DAXX, death domain–associated protein 6; ✓, effects of the variants from the experiments and in silico predictions

Supplementary Table 4. Primer sequences for plasmid construction

Primer	Sequence (5'-3')
H3-3A-G386A-F	AGCTAGCACaCCGCATACGTGGAGAACGTGCT
H3-3A-G386A-R	TATGCGGtGTGCTAGCTGGATGTCTTTTGGCA
H3-3A-C121T-F	ACCTCATtGTTACAGGCCTGGTACTGTGGCGC
H3-3A-C121T-R	GCCTGTAACaATGAGGTTTCTTCACCCCTCCA
H3-3A-G363A-F	aCCAAAAGACATCCAGCTAGCACGCCGCATAC
H3-3A-G363A-R	GCTGGATGTCTTTTGGtATAATTGTTACACGTTTGGCATGG
H3-3A-C365T-F	GCtAAAAGACATCCAGCTAGCACGCCGCATAC
H3-3A-C365T-R	GCTGGATGTCTTTTaGCATAATTGTTACACGTTTGGCA
H3-3A-G271C-F	CAGCTATCcGTGCTTTGCAGGAGGCAAGTGAG
H3-3A-G271C-R	CAAAGCACgGATAGCTGCGCTCTGGAAGCGCA
H3-3A-C166A-F	GACGTTATaAGAAGTCCACTGAACTTCTGATTCG
H3-3A-C166A-R	GGACTTCTtATAACGTCTAATTTCACGGAGCG
H3-3B-A28G-F	AAGCAGACTGCTCGTgAGTCCACCGGTGGGAAAGC
H3-3B-A28G-R	TcACGAGCAGTCTGCTTGGTTCGGGCCATAAG
H3-3B-C23T-F	GCAGACTGtTCGTAAGTCCACCGGTGGGAAAG
H3-3B-C23T-R	ACTTACGAaCAGTCTGCTTGGTTCGGGCCATA
H3-3B-410_411del-F	ggcagtttttatggcgttttgtagGGATCCCGGGTGGCATCC
H3-3B-410_411del-R	acgccataaaaactgccttcacAAGCTCTCTCTCCCCGTATCCG
H3-3B-C68A-F	ACAGCTGGCCAaGAAAGCCGCCAGGAAAAGCG
H3-3B-C68A-R	CTTTCtTGGCCAGCTGTTTGCGGGGGGCTTTC
H3-3B-T155A-F	TCGAGAGAaTCGTCGTTATCAGAAGTCGACCG
H3-3B-T155A-R	AACGACGAtTCTCTCGAAGCGCCACGGTCCCG

Supplementary Figure 1. Intracellular distribution of mutant H3.3 proteins detected in subjects with *H3-3A* mutations.

The cellular nucleus was visualized by DAPI (blue). Both WT and mutant H3.3 proteins (FLAG-tagged, red) accumulated in the cell nucleus. Scale bar, 50μm and 20μm.

Abbreviation: DAPI, 4′,6-diamidino-2-phenylindole; WT, wild type

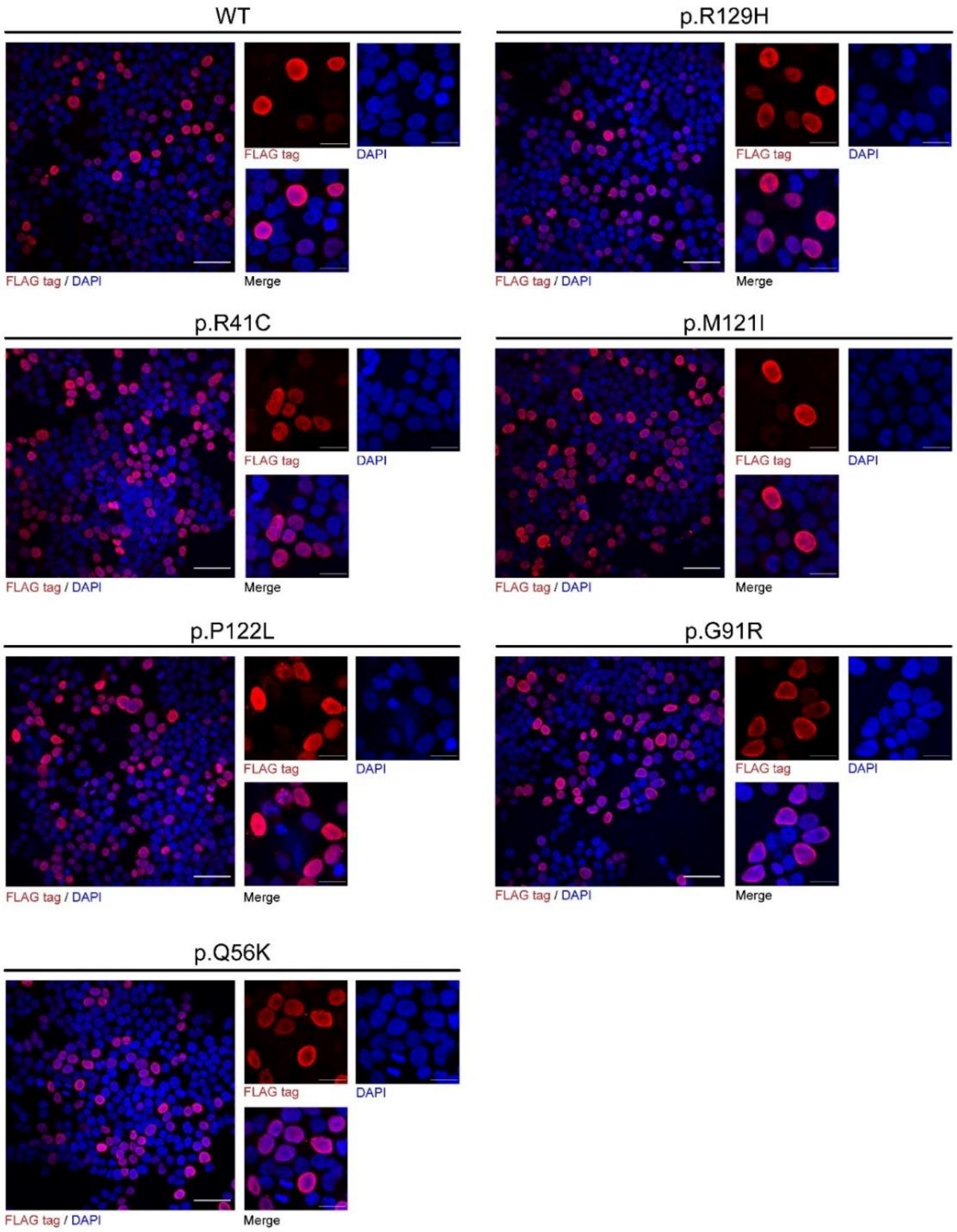

Supplementary Figure 2. Intracellular distribution of mutant H3.3 proteins detected in subjects with *H3-3B* mutations

The cellular nucleus was visualized by DAPI (blue). Both WT and mutant H3.3 proteins (FLAG-tagged, red) accumulated in the cell nucleus. Scale bar, 50μm and 20μm.

Abbreviation: DAPI, 4′,6-diamidino-2-phenylindole; WT, wild type.

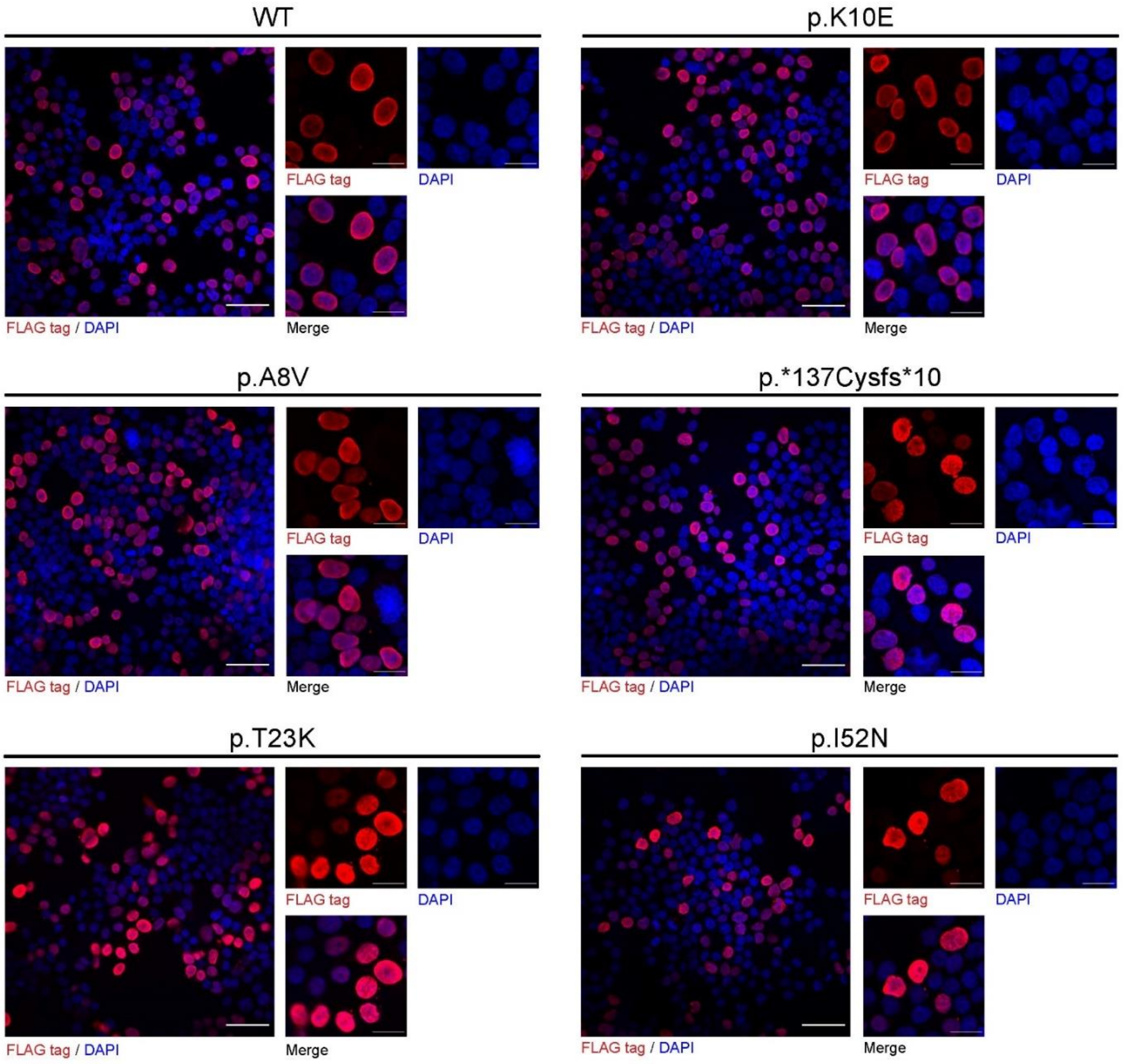

Supplementary Figure 3. *In silico* predictions of post-translational modifications of H3.3 mutants.

Post translational modifications are labelled on the amino acid sequence of histone H3.3. Altered post-translational modifications are indicated by black arrows.

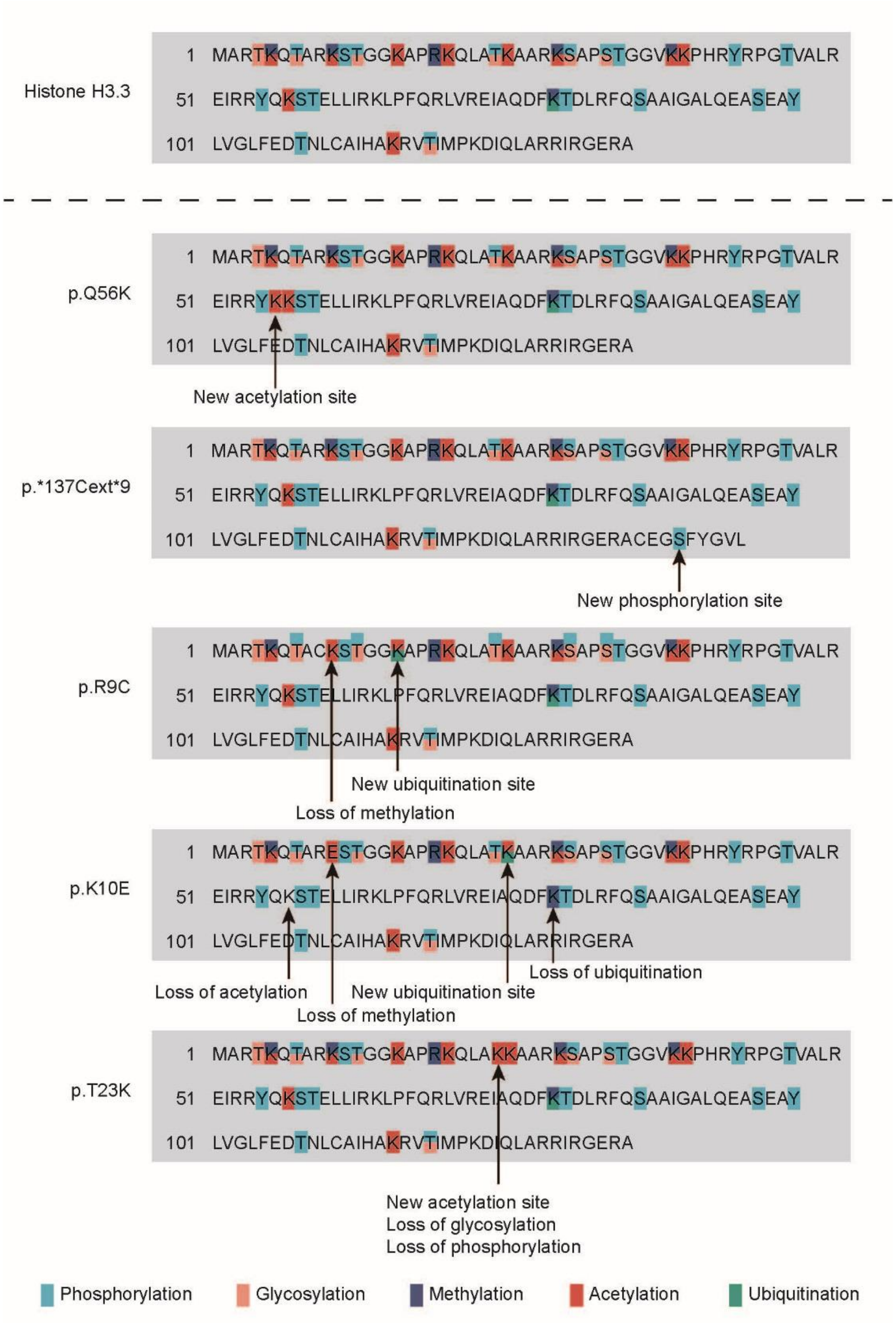

UNPROCESSED ORIGINAL IMAGES

Figure 3a

Variants in *H3-3A*_pFLAG-H3.3

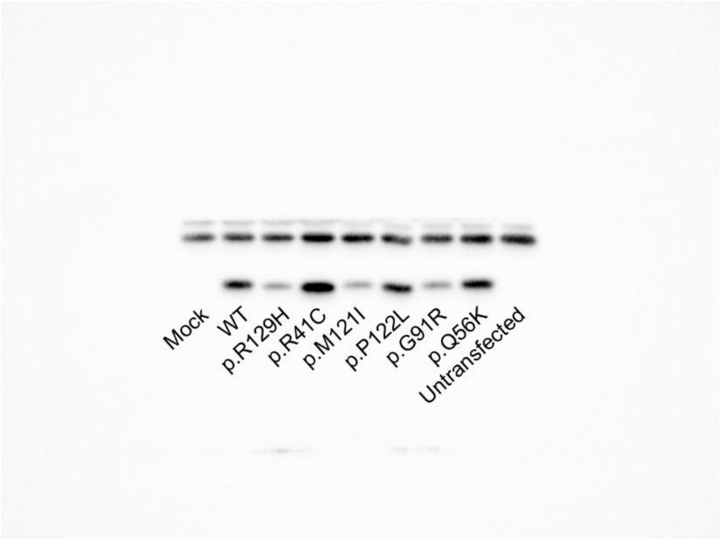

Variants in *H3-3A*_pFLAG-H3.3_Ladder

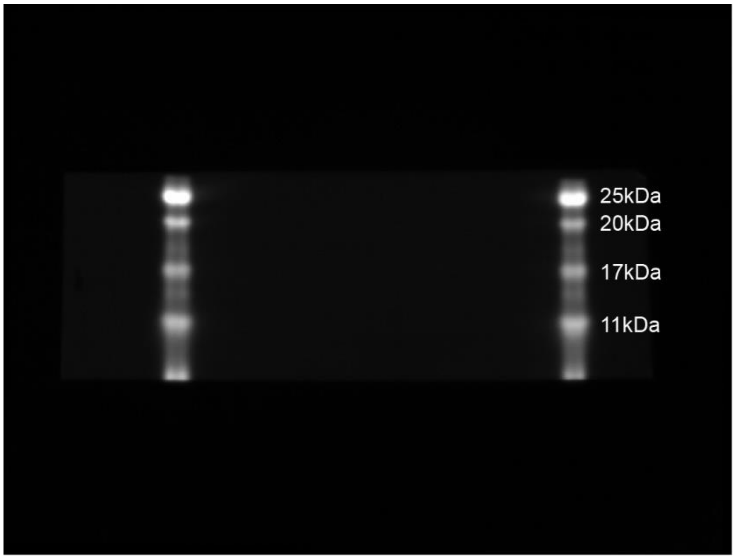

Variants in *H3-3A*_GAPDH

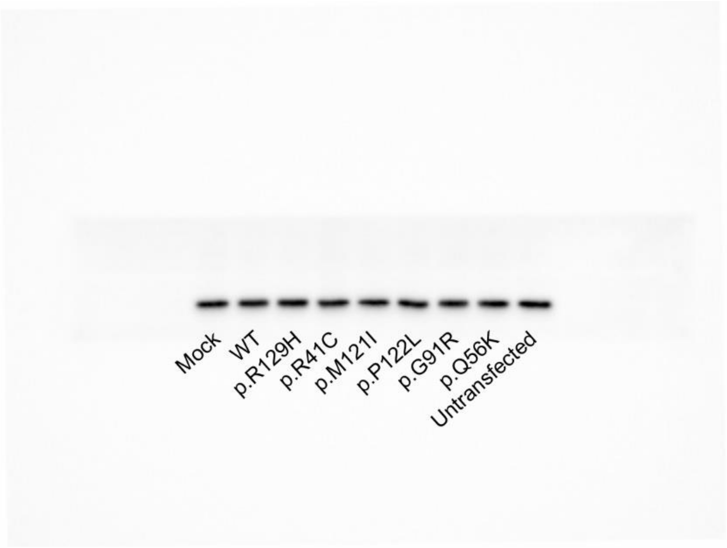

Variants in *H3-3A*_GAPDH_Ladder

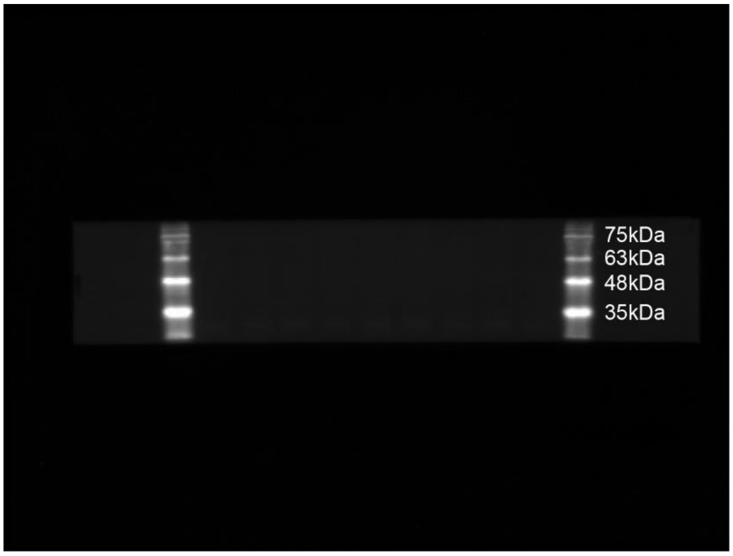

Variants in *H3-3B*_pFLAG-H3.3

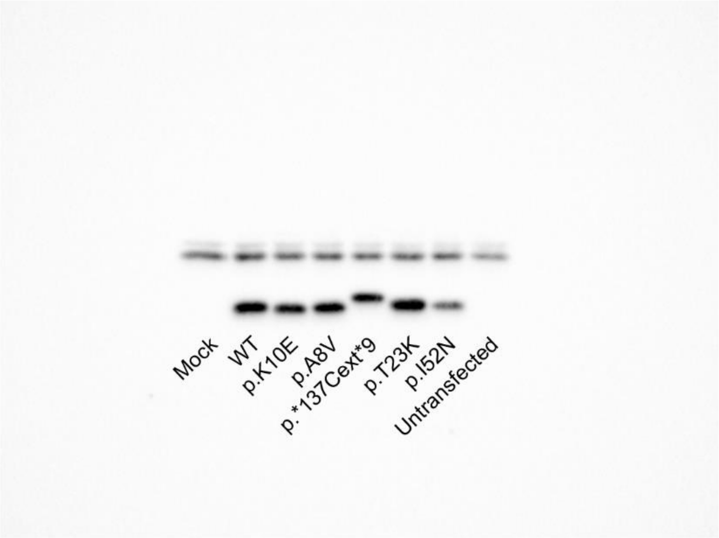

Variants in *H3-3B*_pFLAG-H3.3_Ladder

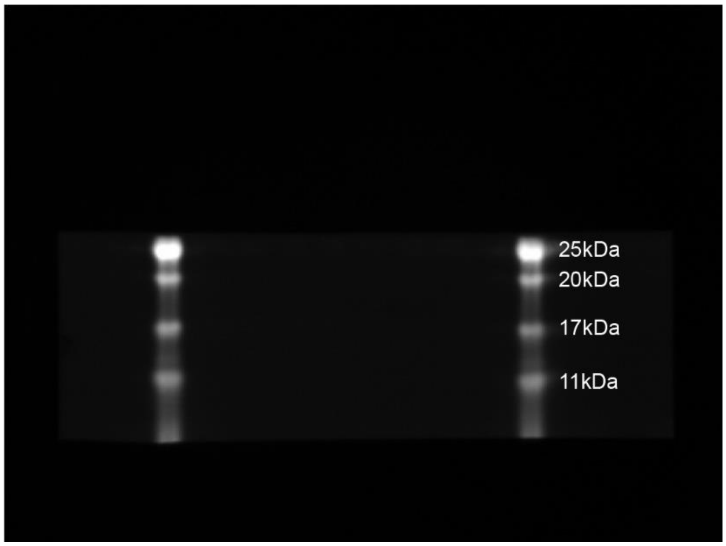

Variants in *H3-3B*_GAPDH

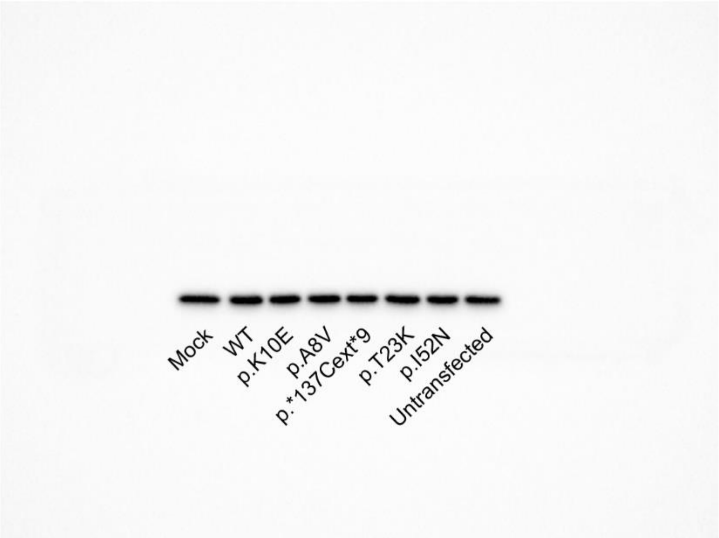

Variants in *H3-3B*_GAPDH_Ladder

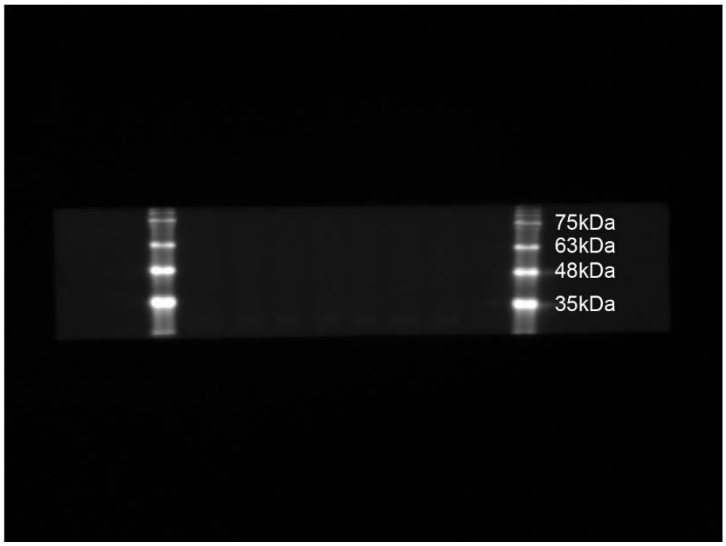

Figure 3c

DAXX_Elution

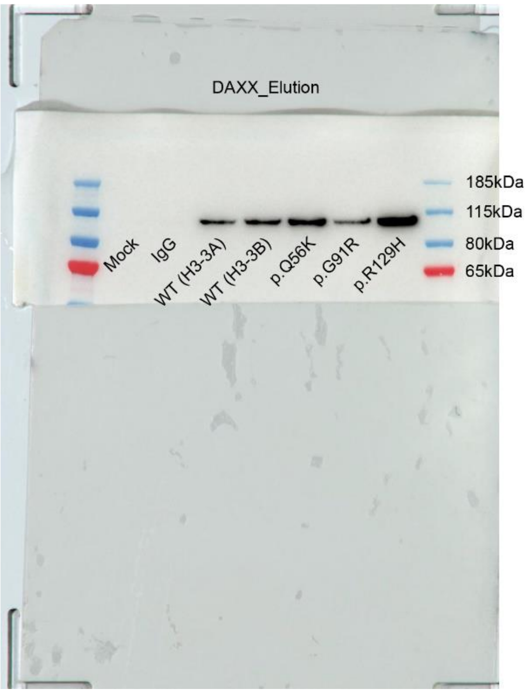

FLAG_Elution

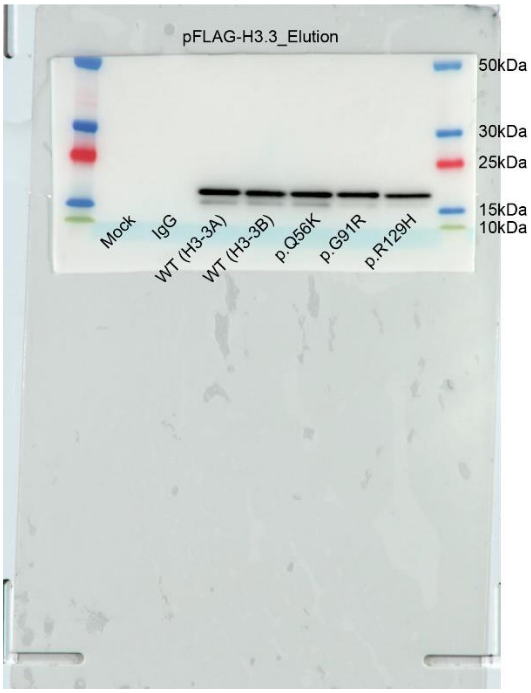

DAXX_Input

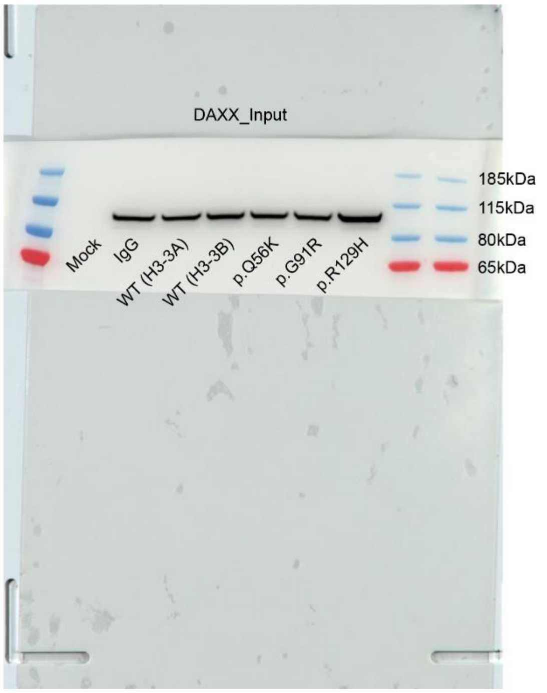

FLAG_Input

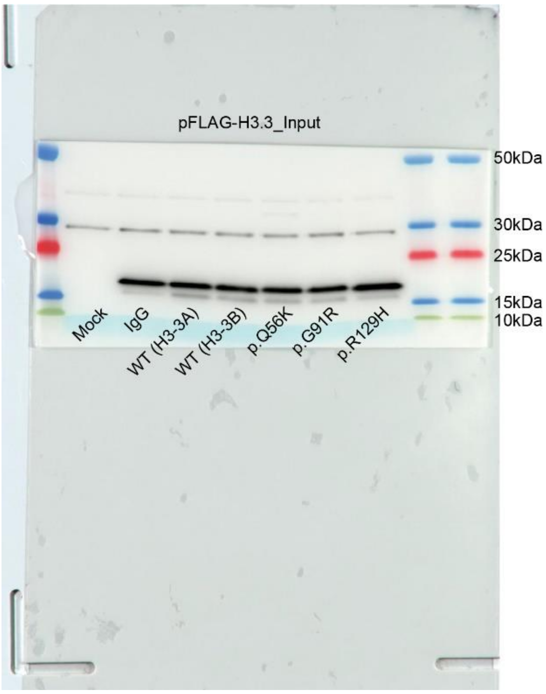

Supplement: Supplementary file 1 — Supplementary Information [file 41525_2021_268_MOESM1_ESM.pdf]
