# Supplementary material for: De novo variants in H3-3A and H3-3B are associated with neurodevelopmental delay, dysmorphic features, and structural brain abnormalities
Source: NPJ Genom Med. 2021 Dec 7;6:104. doi: 10.1038/s41525-021-00268-8 (PMC8651650; doi:10.1038/s41525-021-00268-8)
Supplement: Supplementary file 2 — Reporting Summary [file 41525_2021_268_MOESM2_ESM.pdf]

nature portfolio

Corresponding author(s): Bo Yuan, Nan Wu

Last updated by author(s): Aug 9, 2021

Reporting Summary

Nature Portfolio wishes to improve the reproducibility of the work that we publish. This form provides structure for consistency and transparency in reporting. For further information on Nature Portfolio policies, see our Editorial Policies and the Editorial Policy Checklist.

Statistics

For all statistical analyses, confirm that the following items are present in the figure legend, table legend, main text, or Methods section.

n/a	Confirmed	
☐	☒	The exact sample size (*n*) for each experimental group/condition, given as a discrete number and unit of measurement
☐	☒	A statement on whether measurements were taken from distinct samples or whether the same sample was measured repeatedly
☐	☒	The statistical test(s) used AND whether they are one- or two-sided<br>*Only common tests should be described solely by name; describe more complex techniques in the Methods section.*
☐	☒	A description of all covariates tested
☒	☐	A description of any assumptions or corrections, such as tests of normality and adjustment for multiple comparisons
☐	☒	A full description of the statistical parameters including central tendency (e.g. means) or other basic estimates (e.g. regression coefficient) AND variation (e.g. standard deviation) or associated estimates of uncertainty (e.g. confidence intervals)
☒	☐	For null hypothesis testing, the test statistic (e.g. *F*, *t*, *r*) with confidence intervals, effect sizes, degrees of freedom and *P* value noted<br>*Give P values as exact values whenever suitable.*
☒	☐	For Bayesian analysis, information on the choice of priors and Markov chain Monte Carlo settings
☒	☐	For hierarchical and complex designs, identification of the appropriate level for tests and full reporting of outcomes
☒	☐	Estimates of effect sizes (e.g. Cohen's *d*, Pearson's *r*), indicating how they were calculated

Our web collection on statistics for biologists contains articles on many of the points above.

Software and code

Policy information about availability of computer code

Data collection	The abundance of target relative to control was estimated by densitometry with the ImageJ Software 1.52v.
Data analysis	The one-tailed unpaired Student's t test was used for statistical analysis of immunoblotting results. Statistical analyses were performed with SPSS version 21.0 and GraphPad Prism version 8.0.2.

For manuscripts utilizing custom algorithms or software that are central to the research but not yet described in published literature, software must be made available to editors and reviewers. We strongly encourage code deposition in a community repository (e.g. GitHub). See the Nature Portfolio guidelines for submitting code & software for further information.

Data

Policy information about availability of data

All manuscripts must include a data availability statement. This statement should provide the following information, where applicable:

- Accession codes, unique identifiers, or web links for publicly available datasets
- A description of any restrictions on data availability
- For clinical datasets or third party data, please ensure that the statement adheres to our policy

The reported variants are submitted to ClinVar under the accession numbers from SCV001759938 to SCV001759949.

Field-specific reporting

Please select the one below that is the best fit for your research. If you are not sure, read the appropriate sections before making your selection.

☒ Life sciences ☐ Behavioural & social sciences ☐ Ecological, evolutionary & environmental sciences

For a reference copy of the document with all sections, see nature.com/documents/nr-reporting-summary-flat.pdf

Life sciences study design

All studies must disclose on these points even when the disclosure is negative.

Sample size	We assessed 11 different mutations ascertained from the Baylor Genetics Clinical cohort in HEK293T cells.
Data exclusions	No data has been excluded from the analysis.
Replication	All experiments were performed in triplicate.
Randomization	This is not relevant to the study, because the studied subjects are all from affected individuals and no control cohort has been used.
Blinding	This is not relevant to the study, because the studied subjects are all from affected individuals and no control cohort has been used.

Reporting for specific materials, systems and methods

We require information from authors about some types of materials, experimental systems and methods used in many studies. Here, indicate whether each material, system or method listed is relevant to your study. If you are not sure if a list item applies to your research, read the appropriate section before selecting a response.

Materials & experimental systems

n/a	Involved in the study	
☐	☒	Antibodies
☐	☒	Eukaryotic cell lines
☒	☐	Palaeontology and archaeology
☒	☐	Animals and other organisms
☐	☒	Human research participants
☒	☐	Clinical data
☒	☐	Dual use research of concern

Methods

n/a	Involved in the study	
☒	☐	ChIP-seq
☒	☐	Flow cytometry
☒	☐	MRI-based neuroimaging

Antibodies

Antibodies used	Anti-DDDDK tag (Binds to FLAG® tag sequence) antibody (ab1162; Abcam), Peroxidase AffiniPure Goat Anti-Rabbit IgG (H+L) (111-035-003; Jackson ImmunoResearch), anti-DDDDK antibody (M185-3L; MBL International) , Anti-DAXX antibody (ab32140; Abcam)
Validation	The sources and manufacturers of the antibodies used in this study have been noted in the manuscript.

Eukaryotic cell lines

Policy information about cell lines

Cell line source(s)	A HEK293T cell line was a gift from the Institute of Basic Medical Sciences, Chinese Academy of Medical Sciences
Authentication	The cell line was not authenticated.
Mycoplasma contamination	The cell line was not tested for mycoplasma contamination
Commonly misidentified lines (See ICLAC register)	None

Human research participants

Policy information about studies involving human research participants

Population characteristics	Eleven individuals with de novo variants in H3-3A or H3-3B were identified by retrospective review of ~16,000 individuals referred for clinical exome sequencing (ES) at Baylor Genetics Laboratories (BG) between December 2011 and December 2019 with a range of indications including multiple congenital anomalies and neurodevelopmental disorders. The twelfth individual (Individual 6) was identified through Matchmaker Exchange, thus only clinical and molecular genetics data were presented in the study.
Recruitment	All individuals involved in the study provided informed consents for clinical ES. Inclusion of anonymized individuals with minimum clinical information as part of an aggregated study based on retrospective review of data from BG clinical laboratories was approved by the Institutional Review Board (IRB) of Baylor College of Medicine (BCM, IRB# H-41191). Among the twelve individuals, ten were subsequently recruited into a research study to provide case-level detailed clinical information. These individuals were enrolled by obtaining written informed consents approved by the IRB of BCM (IRB# H-22769). Additional consents for facial photos were also obtained for six of the ten individuals.
Ethics oversight	Institutional Review Board of Baylor College of Medicine

Note that full information on the approval of the study protocol must also be provided in the manuscript.
